# Multifunctional Ultrahigh Sensitive Microwave Planar Sensor to Monitor Mechanical Motion: Rotation, Displacement, and Stretch

**DOI:** 10.3390/s20041184

**Published:** 2020-02-21

**Authors:** Mohammad Abdolrazzaghi, Mojgan Daneshmand

**Affiliations:** Electrical and Computer Engineering Department, University of Alberta, Edmonton, AB T6G 1H9, Canada; mabdolra@ualberta.ca

**Keywords:** double split-ring resonator, displacement, rotation, skew angle, stretch, sensitivity

## Abstract

This paper presents a novel planar multifunctional sensor that is used to monitor physical variations in the environment regarding distance, angle, and stretch. A double split-ring resonator is designed at 5.2 GHz as the core operating sensor. Another identical resonator is placed on top of the first one. The stacked configuration is theoretically analyzed using an electric circuit model with a detailed parameter extraction discussion. This design is first employed as a displacement sensor, and a compelling high sensitivity of 500 MHz/mm is observed for a wide dynamic range of 0-5 mm. Then, in another configuration, the stacked design is used as a rotation sensor that results in a high sensitivity of 4.5 MHz/° for the full range of 0-180°. In addition, the stacked resonator is utilized as a strain detector, and a 0–30% stretch is emulated with a linear sensitivity of 12 MHz/%. Measurements are well in congruence with simulated results, which proves the accurate functionality of the sensor in tracking mechanical deformations, all in a single compact contraption.

## 1. Introduction

Microwave planar sensors have found tremendous applications due to a low cost, non-contact sensing mechanism, and integrability with CMOS/MEMS technology [1,2,3,4,5,6]. The advent of split-ring resonators (SRRs), subwavelength elements inspired by a metamaterial concept, has fostered this trend for its high sensitivity, high quality factor, and compact size [7,8,9,10]. SRRs are shown to perform various types of sensing, including both material characterization and mechanical interference monitoring, using a localized sensitive region that is affected by environmental variations. Examples of this technique include microfluidic chips, integrated with metamaterial-based sensors, to enable lab-on-chip realization [11,12]. Biosensing with a rich history in sensing minute dilutions using optical sensor technology has borrowed metamaterials, supporting confined guided modes, for sensing streptavidin–biotin structure and also for polarization-insensitive material characterization [13,14]. Many material characterizations on solids [15,16], liquids [1,17,18,19,20,21], and gases [22,23,24,25,26] have also been implemented mainly due to high quality factor, subwavelength dimension, and a localized sensitive region to tiny analyte under test at resonance. Metamaterial sensors, overriding traditional radio-frequency structures, offer higher quality factor and lower depth in transmission with respect to the surface tension of the test material [27,28]. Even the newly developed loss-compensated planar sensors using negative resistance removes any concern regarding signal transmission in lossy medium [17,18,20,29,30,31,32]. Another important application of metamaterials is in microwave filter and coupler design that helps shrinking the size while presenting higher performance compared with conventional counterparts [33,34].

Among the many research areas for metamaterial-inspired devices, as stated above, mechanical parameters, such as deformations, displacement, and rotation, are of great interest to be monitored more efficiently and accurately. This is mainly manifested in linear displacements [35] or rotating elements of wristwatches in a small scale. Moreover, on a large scale, turbines are employed as energy harvesting technology [36].

Displacement tracking can be found in RFID barcode reading where resonant-based tags convey unique pieces of information when scanned by a microwave detector [37]. Moreover, microwave rotary sensing platforms act as encoders representing an alternative to optical rotary encoders. Their potential becomes vital when the sensor’s operation at hostile environments (e.g., highly polluted, with high temperature) is required. In a more humane context, light weight and flexible sensors are to complement human motions. The domain for such a low-profile, yet intrinsically flexible, is surging in wearable devices, such as prosthetic hands and wired gloves with artificial intelligence, to report the angular orientation of the joints. This trend has become widespread since conventional methods are limited by the strength of stretch before fatigue [38,39,40]. There is a high need for replacing traditional approaches for mechanical perturbation sensing using optical, for example, optical interferometry, or magnetic elements, for example, Hall-effect encoders, with highly accurate, low-cost, compact, and low-profile methods that are integrable with CMOS/MEMS technology. For such applications, compatibility of the sensor with large-area processing techniques and integrability into flexible devices are paramount to keep up with efforts in regards to expanding a multifunctional apparatus [41,42].

A recent report of one- or two-dimensional displacement sensors are found in [43,44,45] that exhibits the sensing framework based on the notch-depth in a transmission profile. Generally speaking, the notch-depth-based sensing is not intriguing in industrial applications. This is mostly due to the fact that amplitude noise at a low signal level considerably impacts the measurements such that a perfect measurement at a fixed frequency is almost impossible [45]. In addition to displacement, rotation sensing and also stretch analysis are of great importance in detecting mechanical alterations and can be implemented using the same metamaterial-based techniques [45,46,47].

**Figure 1:**
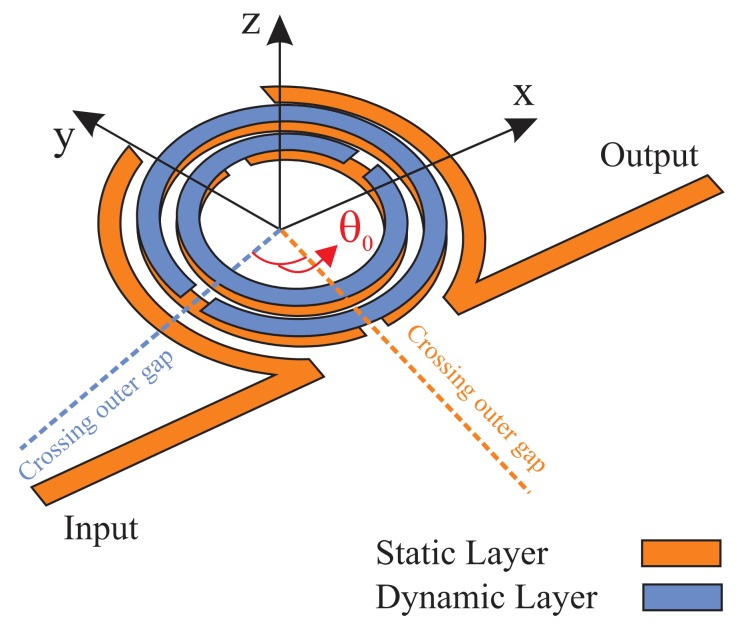
General schematic of the proposed sensor with a depiction of skew angle $\theta_{0}$.

This work proposes a compact multifunctional sensing system comprised of a pair of Double Split-Ring Resonators (DSRR) (as shown in Figure 1). One layer, including a pair of DSRR, is static and is utilized as the reader, while the other one, with electromagnetic coupling, acts as the dynamic layer. Displacement, rotation, or stretch of the dynamic section affects the coupling between these two elements and, hence, is reflected, which is verified in the frequency profile. A theoretical section, first, describes the electrical circuit model and the parameter extraction method for a better understanding of the concept. Next, the effect of the dynamic layer on the first resonator is considered with respect to the strong magnetic coupling. The structure is shown to exhibit much better performance in all sections of displacement (alignment), rotation (skew angle), and stretch compared to the reported literature in terms of the quality factor, sensitivity, and size of the sensor in both simulation and measurement.

## 2. Materials and Methods

Simulations were performed in both High Frequency Structure Simulator ( HFSS) and Advanced Design System (ADS). The presented DSRR elements were fabricated on Rogers 5880 substrate with parameters of $\varepsilon_{r}=2.2$, $\tan\left(\delta \right)=0.0009$, and the stator section has a thickness of 0.8 mm, and the rotary one is either 0.1 or 0.8 mm thick, at two instances. Fabrication includes printing the designs on glossy papers with printing ink and etching the undesired strips of microstrip lines from the surface of the copper-covered substrate. Ammonium persulphate was dissolved in water to etch the copper away.

## 3. Analysis

DSRRs, as the main core of microwave sensors, have shown great functionality due to their compact size and high sensitivity. Here, a DSRR is being loaded with another identical DSRR; hereafter, this combination is called stacked DSRR. In this section, the design parameters of the single DSRRs are discussed first and then is evolved into a stacked one.

### 3.1. Generic Description

**Figure 2:**
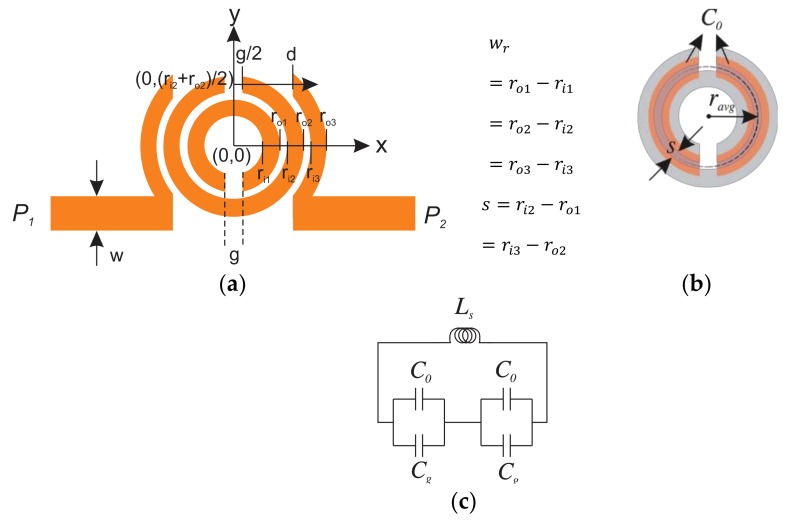
(**a**) Double Split-Ring Resonator (DSRR) dimensions, r_i1 = 1.7,r_o1 = 2.7,r_i2 = 3.1,r_ro2 = 4.1,r_i3= 4.5,r_o3= 5.5,d = 3.5,g = 1,w= 2,w_r = 1,s = 0.4 (all in (mm)), (**b**) capacitive region of the DSRR, (**c**) L-C equivalent of DSRR.

With the dimensions given in the caption of Figure 2, the DSRR is designed on Rogers 5880 substrate ($\varepsilon_{r}=2.2,\tan\left(\delta \right)=0.0009$). The substrate is chosen to be thin (0.8 mm), as the radiation loss is directly proportional to its thickness. The resonator is a combination of the capacitive region between the concentric rings (*C_0_*) (see Figure 2b), gap capacitances (*C­_g_*), and an inductance (*L_s_*) that can be assumed as a transmission line (TL) with an average length of the two rings as:

(1) $$r_{avg}=\;\frac{r_{i1}+r_{o1}+r_{i2}+r_{o2}}{4}=r_{o1}+\frac{s}{2}=r_{i2}-\frac{s}{2}$$

where $r_{i1},\;r_{o1},r_{i2},\;r_{o2}$(given in Figure 2) demonstrate inner/outer radii of inner/outer DSRR, respectively, and *s* is the spacing between them. A combined *LC*-circuit model of the resonator is given in Figure 2c (the parallel resistance associated with the DSRR is ignored for simplicity). 

In the case of complete sensor design, the total capacitance *C_s_* is computed as [48]:

(2) $$\frac{1}{C_{s}}=\frac{1}{C_{0}+C_{g}}+\frac{1}{C_{0}+C_{g}}=\;\frac{2}{C_{0}+C_{g}}$$

The loading TLs are demonstrated with series inductances (*L*) and parallel capacitances (*C_p_*) according to the equivalent circuit of a microstrip TL. The input/output (I/O) TL is coupled to the SRR electrically, which is represented with a pair of equivalent coupling capacitors (*C_c_*). The structure consisting of the two concentric split rings leads to a peak in the transmission profile.

### 3.2. Circuit Model Parameter Extraction

In this section, the values of the circuit model are extracted, and the resultant circuit is simulated in an Advanced Design System (ADS) simulator. For five parameters of interest (*L_s_, C_s_, C_c_, L, C_p_*), five equations are needed to be solved, which are explained as follows.

- Equation for *C_s_*

According to Equation (2), the value of capacitance partly depends on the capacitive region between the two concentric DSRRs, which can be simply computed as follows:

(3) $$C_{0}=l_{\frac{1}{2}ring}C_{pul}=\left(\pi r_{avg}-g\right)C_{pul}$$

where

(4) $$C_{pul}=\beta /\omega Z$$

is the per unit length capacitance of half of the adjacent rings ($l_{1/2ring}$) [48,49] and $r_{avg},\;\beta ,\;\omega ,\;and\;Z$ are average radius (see Figure 2b), phase constant, angular frequency, and impedance of coupled microstrip TL, which is a function of SRR dimensions $w_{r}$ and s. Gap capacitances can be considered as parallel plates with size of $t\times w_{r}$ with t as the copper trace thickness, in distance of *g* from each other. In summary:

(5) $$C_{g}=\varepsilon_{0}t\times \frac{w_{r}}{g}$$

where $\varepsilon_{0}$ is the permittivity of air.

- Equation for *L_s_*

Calculation of total inductance *L* is not that straightforward and approximations are suggested based on variational expression from magnetic energy term; yet, this is pursued using a simpler and still accurate method, as proposed in [50,51]:

(6) $$\neg L_{s}=0.2l\left(2.303\log\left(\frac{4l}{w_{r}}\right)-1.64\right)$$

where *l* is the average length of the rings, which is considered as:

(7) $$\neg l=2\pi r_{avg}-g$$

The resultant inductance will be in (nH).

- Equation for *C_p_*:

In resonance frequency (*f_s_*), the transmission profile would follow the insertion loss associated with the simplified circuit where the series path is short-circuited. The circuit turns into a ­T-model where the central section resembles an impedance parameter:

(8) $$Z_{21}=\frac{1}{j\omega (2C_{p})}$$

This capacitance is due to the microstrip TL-ground and is affecting the transmission value at resonance.

- First Equation for *C_c_*, and *L*

**Figure 3:**
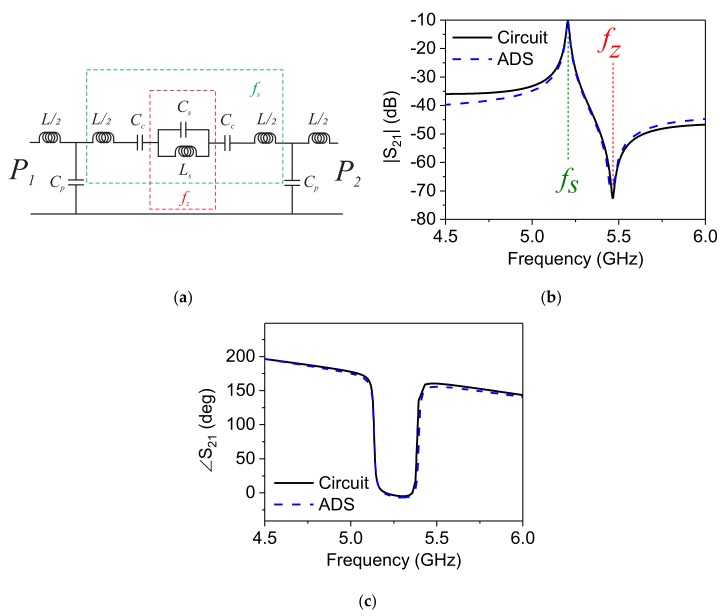
(**a**) LC-circuit model, comparison between Advanced Design System (ADS) simulation and equivalent Circuit model for (**b**) |S_21_ | and (**c**) ∠S_21_ of single DSRR.

Figure 3a presents the full *LC*-circuit considering the coupling between the DSRR and I/O TLs. Resonance frequency (*f_s_*), hence, is where the imaginary of series combination’s impedance (depicted in Figure 3a in green) approaches zero, which can be given as follows:

(9) $$Im\left(\frac{j\omega L}{2}+\frac{1}{j\omega C_{c}}+\frac{\frac{1}{j\omega C_{s}}\times j\omega L_{s}}{\frac{1}{j\omega C_{s}}+j\omega L_{s}}+\frac{1}{j\omega C_{c}}+\frac{j\omega L}{2}\right)\to 0$$

- Second Equation for C_c_ and L

Another equation is required to resolve all of the parameters. It is derived from the dispersion characteristic of a periodic structure consisting of unit cells (shown in Figure 2a), as given below [52]

(10) $$\cos\phi =1+\frac{Z_{s}\left(\omega \right)}{Z_{p}\left(\omega \right)}$$

with *Z_s_* and *Z_p_* being series and parallel impedances of the π-model of the equivalent circuit. At the frequency of $f_{\pi /2}$ where $\phi_{S_{21}}=90$, this condition reduces to a simpler form of

(11) $$Z_{s}\left(f_{\frac{\pi }{2}}\;\right)=\;-Z_{p}\left(f_{\frac{\pi }{2}}\;\right)$$

Hence, all of the parameters can be extracted uniquely based on purely analytical formulae and the transmission profile from the simulated design. The proposed DSRR is simulated in ADS, and its equivalent circuit model is extracted using the previous equations. The results in Figure 3 demonstrate that both magnitude $\left|S_{21}\right|$ and phase $∠\;S_{21}$ of the *LC*-circuit model agree with the simulation. The parameter values are populated in Table 1.

It should be noted that *f_s_* (see Figure 3b) is the resonance frequency of the sensor that is extracted, as mentioned earlier in this section. However, *f_z_* is also important as known as the transmission zero of the sensor:

(12) $$f_{z}=\frac{1}{2\pi \sqrt{L_{s}C_{s}}}$$

### 3.3. Stacked Resonator

**Figure 4:**
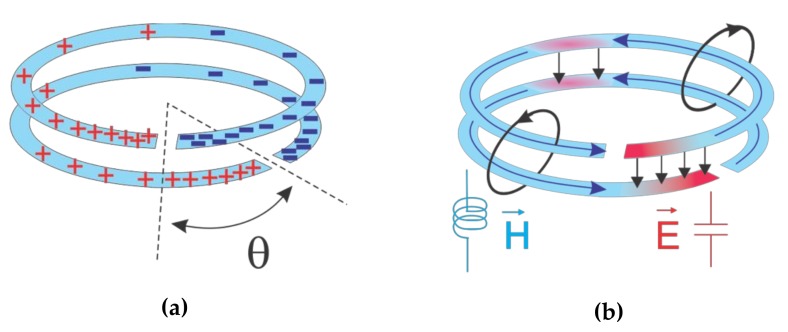
(**a**) Distribution of charges on SRRs in stacked resonator, (**b**) establishment of E/H fields equivalent to C_s_/L_s._

**Figure 5:**
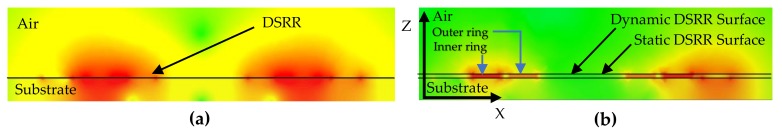
Electric field distribution of (**a**) single- and (**b**) stacked-DSRR in a similar ZX cross-section.

In this section, the basic DSRR is exploited as the core of a stacked combination of two identical resonators, as shown in Figure 4 (only outer rings are sketched for simplicity). The purpose of this design is to analyze mechanical disturbances, including rotation, displacement, and stretch. In order to elicit mechanical interactions, at least two separate sectors should be implemented: static and dynamic. In the proposed design, a static resonator is assumed to be the reader and is connected to the measuring device; here, a vector network analyzer (VNA). The dynamic resonator, consisting of a DSRR pattern on groundless substrate, introduces external disturbance to the electromagnetic field distribution of the static resonator. Dimensions and properties of both resonators are identical, but the dynamic one is implemented on thinner substrate (0.15 mm), which is supposed to be moving as the top layer. Its orientation with respect to the lower DSRR is defined by the angular separation between the gaps, denoted as skew angle $\theta_{0}$, as shown in Figure 4a. On the other hand, similar charges can flow in the same direction since they share the same magnetic flux (magnitude and direction) and produce a similar magnetic field H (see Figure 4b). According to Figure 5, it can be inferred that the E field is mainly confined within the region between the two gaps (red spot in Figure 5b. This illustrates the origin of capacitance and inductance being formed as a result of high magnetic coupling between the stacked (broadside coupled) DSRRs.

**Figure 6:**
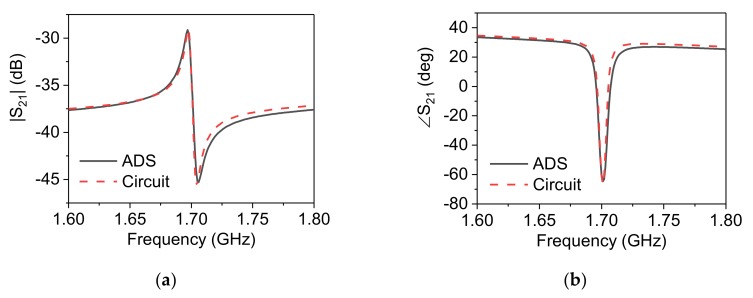
Comparison between ADS simulation and equivalent Circuit model for (**a**) ­$\left|S_{21}\right|$ and (**b**) $∠S_{21}$ of stacked DSRR.

The extraction of single DSRR parameters can be similarly applied to the stacked DSRR, with a major change in the capacitance of the resonator. In addition to the initial capacitance between concentric split rings of the static resonator, not only the distance between two DSRRs in stacked configuration reduces to the thickness of the dynamic resonator’s substrate (thickness = 0.15 mm) but this mutual capacitance is also supported by a much larger overlapped area between these metallic DSRRs. This overlap starts growing from $\theta_{0}=0$ and, in the case of $\theta_{0}=180{}^{\circ}$, brings about maximum coupling, which results in a drastically lowered resonance frequency of 1.7 GHz, as shown in Figure 6. Moreover, the circuit parameters are extracted using the same method as stated in Section 3.2., except for the capacitance that has an additional portion following a parallel plate capacitance scheme (this is developed in Section 4. 2) and is also given in Table 1. A great match between the simulation of the sensor in both the ADS and circuit model is represented in Figure 6 for both amplitude and phase.

## 4. Simulation and Measurement

In this section, different states of the dynamic DSRR are examined with respect to static DSRR, and the functionality of the device can be elaborated when being employed as a rotation/ displacement/stretch detector. In order to analyze the sensor within the stated conditions, first, the relationship between the two identical resonators in the vertical distance is studied.

### 4.1. Vertical Separation

**Figure 7:**
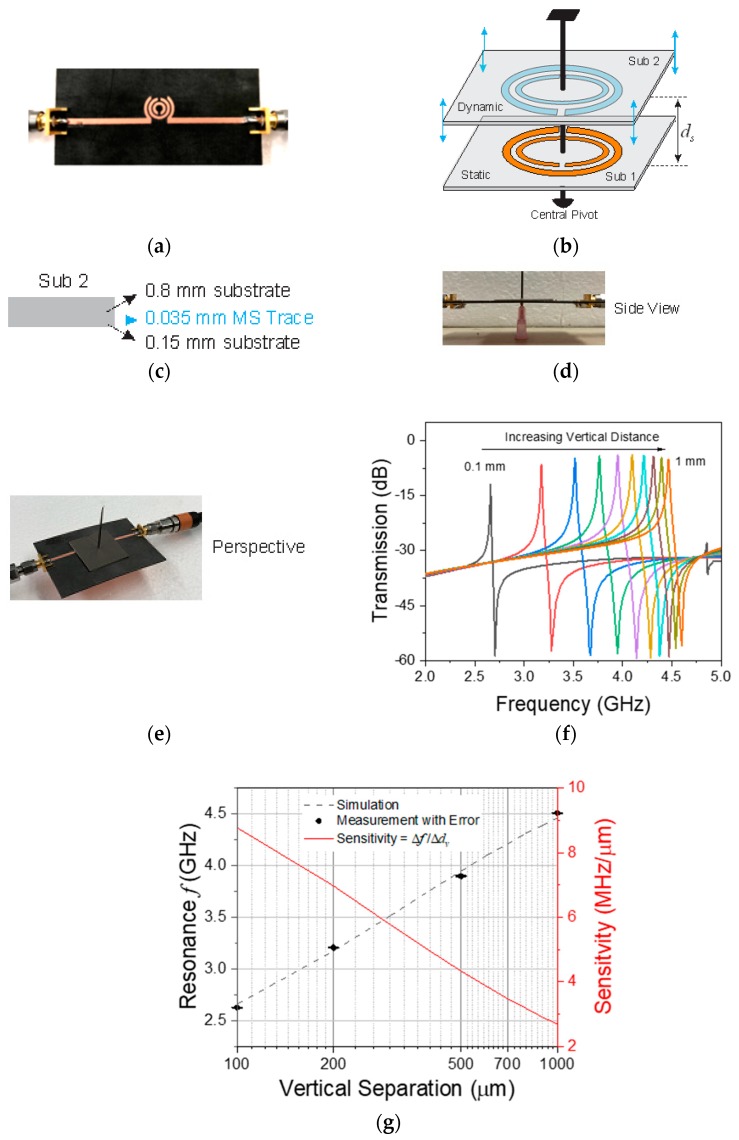
Fabricated static sensor, (**b**) schematic of vertical displacwment of the dynamic DSRR, (**c**) details of sub-2, measurement in (**d**) sideview, and (**e**) perspective, (**f**) transmission profile of the simulated sensor with respect to vertical separation, (**g**) agreement between simulation and measurement results on vertical displacement.

The loading impact of the coupled resonators on each other increases as they become closer in proximity and the coupling between them enhances. This behavior is examined to extract the extreme level of mutual interaction. The sensor is fabricated on Rogers 5880 substrate with the dimensions given in the caption of Figure 1, as represented in Figure 7a. The dynamic DSRR is configured with a twist angle of θ=180° (see Figure 4a) for the maximum interaction (which will be discussed in the next section in more detail) and is swept vertically along a central pivot, which supports both resonators (Figure 7b). The dynamic resonator is supported with another thicker substrate of the same property with the configuration given in Figure 7c. A fabricated sensor is also shown in both side- and perspective view in Figure 7d,e. The swept distance ranges between ranges within *d_s_* = 0.1 –1 mm with increments of 0.1 mm in ADS simulation. The resultant resonance frequency reduces as the distance (*d_s_*) becomes smaller, and this behavior is nonlinear even for *d_s_* < 1 mm. The downshifts in the main resonance frequency are apparent in Figure 7f to follow logarithmic behavior and are highly sensitive for the smallest variations in the μm range. These simulations are validated using Cobalt C2420 VNA, and a great agreement is observed between the measured values and simulated structure, as shown in Figure 7g with an embedded sensitivity curve, which also takes nonlinear form yet averages at S ~ 6 MHz.μm^−1^ when $S=\Delta f/\Delta d_{v}$.

### 4.2. Rotation Analysis with Skew Angle $\theta_{0}$

The static and dynamic resonators form a parallel capacitance that is related to the physical dimension of the strips, their distance, and the dielectric slab between them. This capacitance is the core element of this section’s analysis since the dynamic resonator is assumed to be placed on the static resonator, and its varying orientation is determined with skew angle $\theta_{0}$.

**Figure 8:**
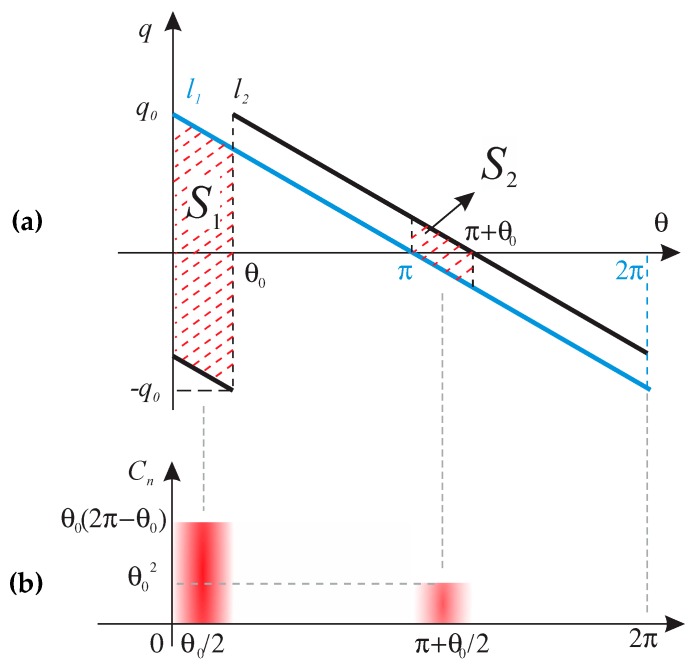
(**a**) Conceptual charge distribution on the two stacked SRRs (l_1_,l_2_), (**b**) normalized capacitance C_n_ (or E-field concentration) on SRR based on charge distribution.

Charge distribution is intense at the edges close to the gap and nulls at the center of each split ring, as mentioned before. This can be simply considered as a linear charge distribution regarding skew angle on the surface of microstrip resonators. Charge distribution on each SRR can be defined as follows:

(13) $$q_{1}=q_{0}\left(\pi -\theta \right)/\pi$$

(14) $$q_{2}=q_{0}\left(\pi -\theta +\theta_{0}\right)/\pi$$

where *q*_0_ acts as the maximum value. Figure 8a demonstrates this behavior on two overlapping SRRs with a skew angle of $\theta_{0}$ between them with an assumption of infinitesimal gap size to only grasp the concept. Following linear charge dispersion, hatched sections in Figure 8a contain opposite charges facing each other and are responsible for the capacitance of the net summation at zero. One can calculate the total capacitance by integrating it over the area of interest with respect to the skew angle $\theta_{0}$ within the region of $\theta_{0}\in \left(0:\theta_{0}\right)\cup \left(\pi :\pi +\theta_{0}\right)$ (see Figure 8b):

(15) $$C_{overlap}=\varepsilon_{0}\varepsilon_{r}\left(\int_{0}^{\theta_{0}}\frac{dA}{d_{s}}+\;\int_{\pi }^{\pi +\theta_{0}}\frac{dA}{d_{s}}\right)=\;\varepsilon_{0}\varepsilon_{r}\theta_{0}\left(r_{o2}^{2}-r_{i2}^{2}\right)$$

Equation (15) denotes that the capacitance of the system due to the positioning of opposite charges in front of each other is linearly proportional with the skew angle $\theta_{0}$, and the overlapped region imposes an additional capacitance increase.

There is a similar trend, but in the opposite direction, for *L_s_* that can be easily verified using the same analogy explained above considering regions of $\theta_{0}\in \left(\theta_{0}:\pi \right)\cup \left(\pi +\theta_{0}:2\pi \right)$, while replacing the current/H-field distribution with the charge/E-field distribution.

The total capacitance is:

(16) $$C_{total}=C_{overlap}+\frac{C_{0}+C_{g}}{2}\;≅C_{overlap}\propto \theta_{0}$$

where $C_{g}\;\mathrm{and}\;C_{0}$ are computed from Equation (2). The total capacitance is mainly determined by the parallel plate capacitance between the overlapping SRR traces since the DSRR-separation is much smaller than the wavelength ($0.15\;mm\;\sim \;3.6\times 10^{-3}\lambda_{g}$), where $\lambda_{g}$ is the guided wavelength in the substrate.

The inductance is also impacted by the disturbed current flow in DSRRs with respect to the skew angle $\theta_{0}$, following a similar analysis as mentioned for the capacitance.

**Figure 9:**
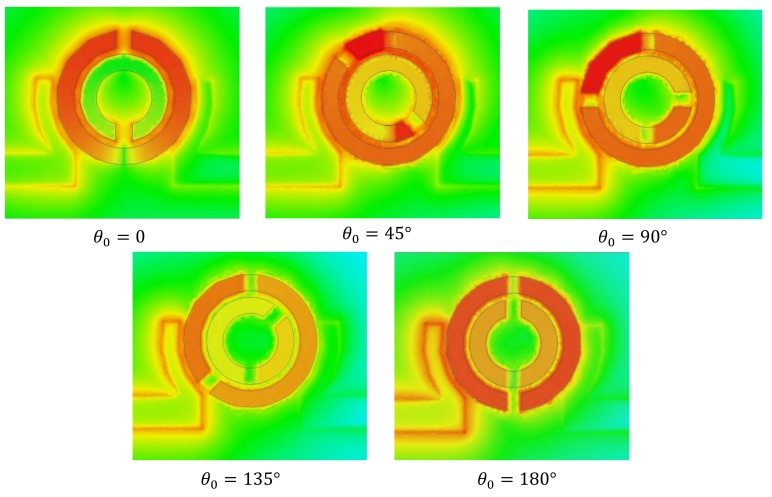
Electric field distribution of the stacked DSRR with various skew angles of 0, 45, 90, 145, and 180 deg, simulated with HFSS.

The electric field distribution on the XY-surface at Z = 0.075 mm between two DSRRs at Z = 0, and 0.15 mm is demonstrated in Figure 9. The highest overall concentration of E-fields at $\theta_{0}=180{}^{\circ}$ is apparent from this figure, and since mainly the electric field is confined between the rings instead of spreading out (see Figure 5b), the stacked DSRR approaches a closed waveguide in which its length is determined by the skew angle and is more pronounced on the outer ring.

**Figure 10:**
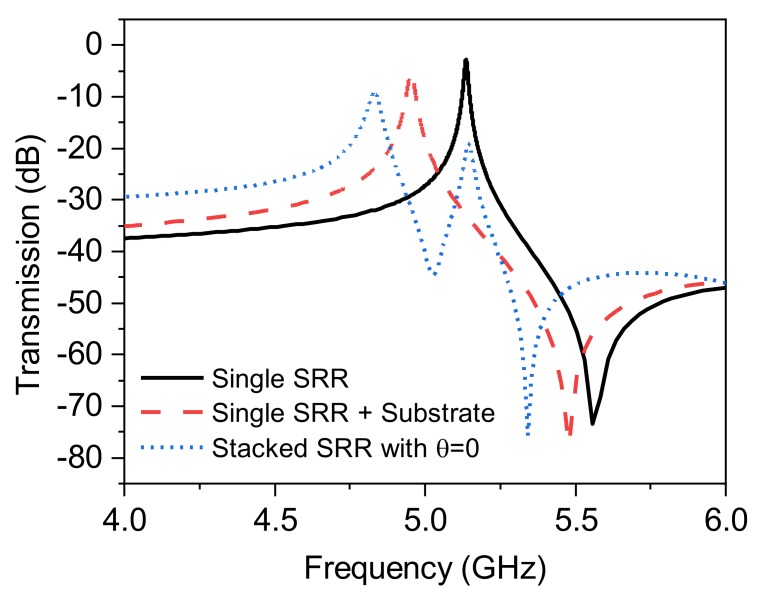
Measured comparison between single DSRR, its loading with substrae, and its stacking with a DSRRat $\theta_{0}=0{}^{\circ}.$

Since the capacitance, in essence, is tightly linked to the separation of an equal number of opposite charges, such condition is not observed in $\theta_{0}=0$. This concept, as shown in Figure 9, suggests that the case of zero skew angle is not benefited from stacked DSRR, opposite charge aggregation, and the resultant extra capacitance. A verification of this is given in Figure 10, where a comparison is shown between a normal DSRR and a loaded one with another aligned DSRR ($\theta_{0}=0$). The amount of downshift in frequency, which is a function of introduced capacitance, is ~300 MHz, shown as the dotted line. In order to extract the effectiveness of this loading, a configuration of static DSRR loaded only with a similar, but copper-free, substrate (thickness and permittivity) as dynamic DSRR is also measured, shown with a dashed line. A shift of ~180 MHz is achieved, which is a pure effect of the substrate loading. It can be inferred that a major impact of the downshift in the aligned case ($\theta_{0}=0$) is not from the stacked configuration and its extra capacitance, but from substrate, where the overall downshift is negligible.

**Figure 11:**
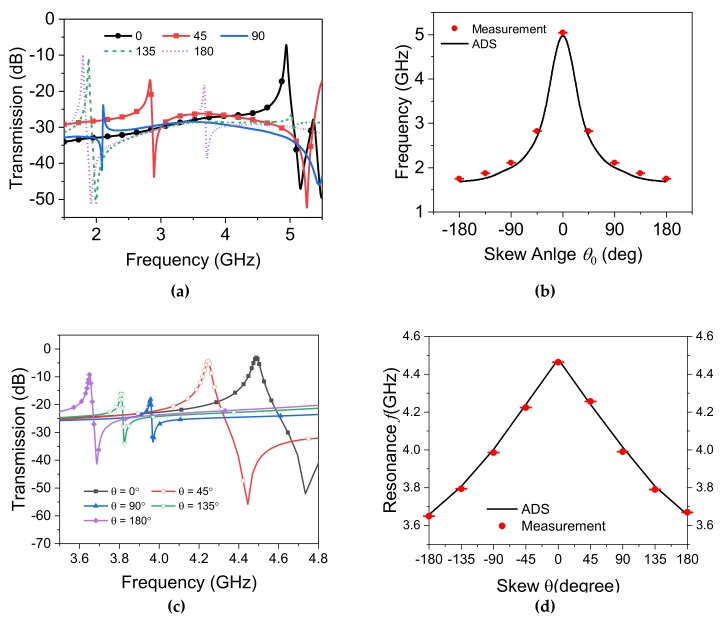
(**a**) Measured transmission profile variation due to rotation with the skew angles of 45, 90, 135, and 180 degrees, (**b**) comparison between simulation and measurement data for skew angle analysis, (**c**) measured transmission profile for thicker rotary DSRR on 0.8 mm substrate, (**d**) comparison between simulation and measurement for the modified sensor on thicker rotary DSRR.

Linear addition of capacitance upon skew angle change results in a resonance frequency shift as $\propto 1/\sqrt{\theta_{0}}$. It can be inferred from *f_z_* given in Equation (12) with respect to the fact that fs is linked with *f_z_* due to the loading capacitance Cc. Confirmation of this assertion is pursued by measuring the dynamic DSRR’s rotation on top of the static DSRR (see Figure 11a). The strongest variations in the resonance frequency result when the whole structure’s symmetry is broken at lower skew angles. The maximum shifts occurs at $\theta_{0}=180$, following maximum modification in capacitance and inductance. 

For quantitative comparisons, the sensitivity can be defined as follows:

(17) $$S_{\theta }=\frac{{\Delta f}_{res}}{\Delta \theta }$$

which is the rate of change in the sensor’s output (here frequency) based on unit variation in the parameter under test (here, skew angle), and this is represented with the dimension of [Hz/­°]. A significant frequency reduction as much as three times from $\theta_{0}=0{}^{\circ}\;:180{}^{\circ}$ showcases a highly strong coupling that is translated into an average sensitivity of $S_{\theta }\approx 18.9\;MHz/{}^{\circ}$. Maximum resonance frequency reduction aligns with the maximum extra loading capacitance from a dynamic resonator. It is also seen that the amplitude of the transmission is significantly lowered at skew angles around $\theta_{0}=90{}^{\circ}$, a result of symmetry breakdown that limits the maximum coupling between I/O TLs and the stacked DSRR combination. A simulation of similar design is performed in ADS, which offers more data points for a comprehensive grasp of the trend in resonance frequency and its dependency on the skew angle. A great agreement between the simulated and measured data proves satisfactory accuracy of the simulations in Figure 11b.

### 4.3. Rotation Sensing With Wide Dynamic Range 

It is noteworthy that this specific configuration is ultra-sensitive to the rotation that enters nonlinearity when the variations exceed $\theta_{0}≅50{}^{\circ}$. This can be useful for applications involving small drifts in the alignment of the two concentric DSRRs. The amount of the coupling determines which region the sensor behaves in. In order to enhance the functionality of the sensor in terms of dynamic range and enabling applications with high degree of rotation, the design can be slightly modified to obtain linear performance throughout the skew range of 0 to±180°. In this case, the loading between the two opposite-DSRRs is qualified with larger substrate thickness on the rotary one to move the second DSRR slightly away from the static DSRR and reduce the coupling. This has resulted in a fully linear sensor at the expense of reduced, and still high, overall sensitivity. Figure 11c demonstrates the transmission response of the sensor when the rotary DSRR is established on 0.8 mm thick substrate rather than 0.1 mm, and the measured verification is given in Figure 11d. This wide dynamic range translates into sensitivity of $S_{\theta }\approx 4.5\;MHz/{}^{\circ}$.

### 4.4. Horizontal Displacement 

**Figure 12:**
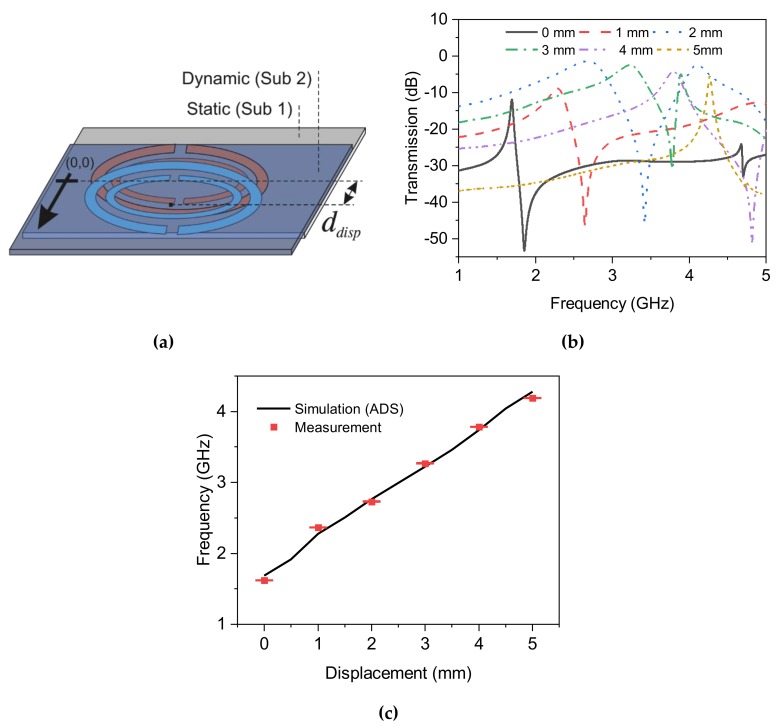
(**a**) Schematic of horizontal displacement of the dynamic resonator with respect to the static one, (**b**) measured transmission profiles for horizontal displacement up to 5 mm, (**c**) comparison between measurement and simulation.

In this section, the sensor is exploited to determine minute displacements in a mechanical system. To this end, the same dynamic resonator on thin substrate is placed on top of the static resonator in the opposite direction (see Figure 12a). This can be considered as a skew angle of $\theta_{0}=180{}^{\circ}$, which results in maximum interaction. As the centers of the two DSRRs separate as much as *d_disp_*, the coupling between them reduces, and due to the circular shape of the structure, this reduction is linear. The whole separation takes place with identical steps in frequency upshifts. Displacements of 2 mm and 3 mm, as shown in Figure 12b, brings about lower coupling between individual DSRRs. Please note that the DSRRs are not at the same condition since the static one has a larger thickness and is also grounded, while the dynamic one is only supported with extremely thin substrate, thus the trend in their coupling effect is not assumed to be similar. It is noted that the quality factor of the transmission profile for these cases is remarkably reduced because each DSRR is loaded with another one where the electromagnetic energy that has been encapsulated between the traces (as in $\theta_{0}=0{}^{\circ}$) now propagates into free space, and hence, the stored energy drops significantly. 

Figure 12b also illustrates a more comprehensive frequency shift with respect to intermediate values of displacement. The linear trend is fitted with the expression as follows:

(18) $$f_{res}=1.73+\frac{d_{disp}}{2}$$

that with the definition of sensitivity as:

(19) $$S_{disp}=\frac{\Delta f_{res}}{\Delta d_{disp}}$$

suggests a sensitivity of ~500 MHz.mm^-1^, an extremely high performance that overpasses the current devices in very recent literature with a reported sensitivity of 207.2 MHz.mm^-1^ [53]. This linear relationship is extended to the full span that was available for the outer radius of the outer SRR; hence, in order to enhance the range of interest, the radius of resonators should be increased that leads to a sensor resonating at lower frequencies. On the other hand, this ultra-high sensitivity allows reducing the minimum achievable separation down to 2 μm, known as the limit of detection, since the minimum frequency variation in the readout of this resonator is measured to be 1 MHz at best.

### 4.5. Strain Sensing 

**Figure 13:**
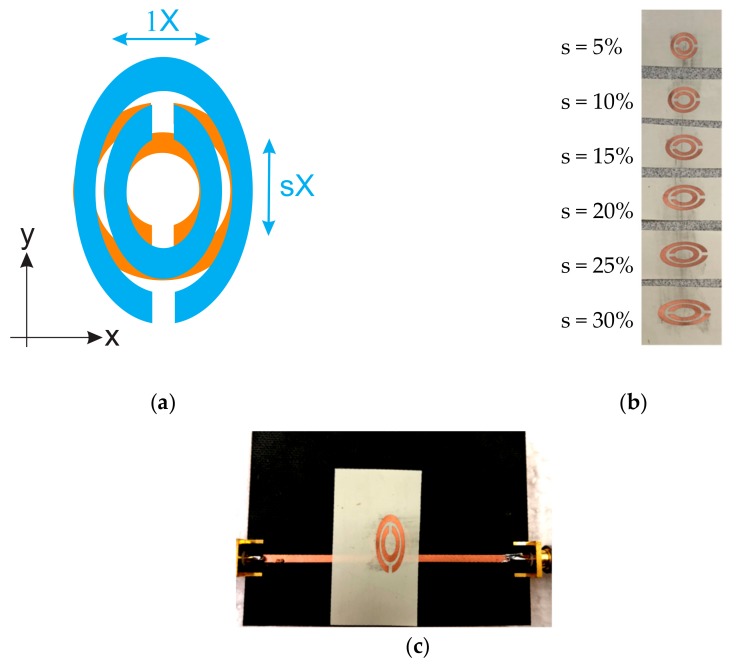
(**a**) Schematic of scaling concept, (**b**) fabricated scaled dynamic DSRRs ranging from 5–30 %, (**c**) measuring scaled DSRR as stretched.

**Figure 14:**
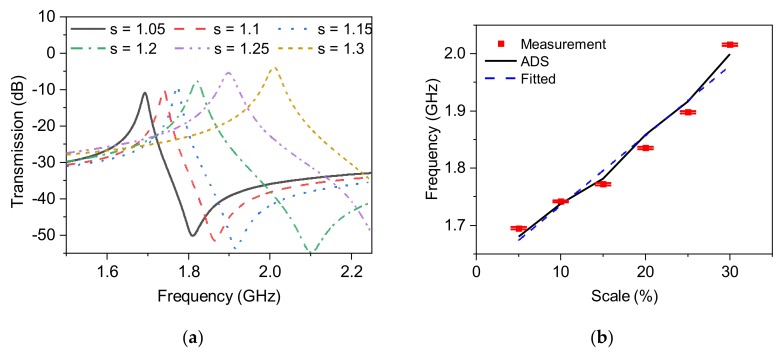
(**a**) Upshifts due to the scaling behavior in dynamic DSRR, (**b**) comparison between the simulated and measured results of scaling.

In this section, another feature of mechanical perturbation is discussed, namely, a stretch in materials. This is usually observed in elastic materials but is also important in solids to prevent a rupture in the texture of hard substances. In this part, ideally, an elastic element should be utilized as the dynamic section of the sensor. However, in a more simplified, yet more stable and repeatable experiment, instead of stretching a normal substance, an already-stretched material is emulating the dynamic section. More specifically, the same dynamic DSRR is expected to be used as the stretchable element with a factor of s (see Figure 13a). As long as the substrate is not stretchable and printing the desired pattern on elastic substrate is of a challenge that is out of feasible available fabrication facilities for proving the concept, the same substrate (thickness and permittivity) is patterned with stretched DSRRs, as shown in Figure 13b. The range in which the dynamic DSRR is stretched in only Y-direction varies from 5–30%. The installation of the dynamic DSRR on the static DSRR is illustrated in Figure 13c, and the transmission profile is recorded in each step towards divergence so that the exposure of common areas on SRRs facing each other become less; hence, the generated equivalent capacitance reduces. This trend results in upshifted resonance frequencies, as shown in Figure 14a. An apparent reduction in the quality factor of the resonances is clearly observed with increasing the stretch ratio (s). This is similar to the concept mentioned in Section 4.3, where the stored energy is reduced as it propagates into free space and no longer impacts for 30% stretch and fills the intermediate region, as shown in Figure 14b. This great agreement between simulation and measured results is also fitted with a linear function as follows:

(20) $$f_{res}=1.65+0.012\;s$$

where *s* is the stretch factor in %. A sensitivity of 12 MHz per 1% stretch is achieved, resulting in the limit of detection of *s* = 0.085% with the assumption that the minimum frequency variation to be reliably detected is ~1 MHz.

### 4.6. Comparison of the Reported Literature

In this section, a conclusive comparison is presented between the proposed sensor and the reported literature that are somehow related to sensing/detecting mechanical perturbations.

It is noteworthy to mention that this paper proposes unique capabilities of a single design to be used in monitoring almost all mechanical perturbations: rotation, displacement (alignment), and strain. Having said that, the reported literature only contains one of these features given in detail in Table 2.

Displacement sensing, also known as alignment sensing, is reported by two detection methods of amplitude- or frequency tracking. In the former, which is based on symmetric magnetic flux flow through the resonator, resonance is established at a fixed frequency, and as a result of displacement in the dynamic part of sensor, the symmetry breaks down, and a change in the depth of notch occurs [44,45]. A sensing unit is then defined based on the amount of change in the depth of *S*_21_, as dB/mm. These sensors present high sensitivity ($\partial \left|S_{21}\right|/\partial d_{disp}$) with low dynamic range (${\left|S_{21}\right|}_{max}-{\left|S_{21}\right|}_{min}$) as a huge sensitivity of 65 dB/mm in [44] is just limited by a 0.3 mm displacement, which is not applicable to so many practical applications. On the other hand, sensing based on amplitude is more prone to the background noise and, hence, is not a reliable method for highly sensitive applications. In the latter method of sensing (frequency-based), the resonance frequency of a design undergoes variation as the dynamic part moves, similar to what is elaborated in this manuscript. Low sensitivity with high dynamic range is of inherent features of this method. The dynamic range in [53] is highly improved up to 5 mm with a reported ultra-high sensitivity of 207 MHz/mm. However, the proposed sensor, in this study, is offering way more sensitivity of 500 MHz/mm for the same dynamic range with a much smaller electrical size of the sensor.

The same line of thought of amplitude measurement also applies for rotation sensing, where asymmetry brings about variation in the depth of transmission profile [46,54]. In these cases, in order to have relatively high sensitivity (1.87 dB/°), one must add to the electrical size considerably more than the guided wavelength in the substrate ($\lambda_{g}$) [54]. The proposed sensor, with a more reliable frequency-detection based sensing scheme, offers remarkably high sensitivity of 4.5 MHz/°, which is measurable with high accuracy and stability.

The authors of [47] demonstrated a stretchable sensor based on complementary SRR with 24 MHz/% measured sensitivity. This high value came at the cost of a very low quality-factor of 4.5–4.8 and large electrical size of 2.5$\lambda_{g}\times \;$3.5$\lambda_{g}$, while the proposed sensor exhibits a high Q-factor of 226 with a very small size of 0.13$\lambda_{g}\times \;$0.13$\lambda_{g}$ and a simpler design.

All in all, this work has the unique feature of gathering all of the capable functionalities of a sensor to monitor many mechanical disturbances into a single system with high quality factor, high linearity, and extremely high sensitivity.

## 5. Conclusions

This paper presents a two-element microwave planar sensor, namely, a double split-ring resonator design, resonating at 5.2 GHz, which is accompanied by another identical pattern on a groundless thinner substrate. This single sensor is configured in three different setups to enable measuring mechanical variations of displacement, rotation, and stretch. The equivalent circuit model for only single SRR and also stacking the two resonators is presented with great agreement between ADS simulations. Stacking these two elements is used for sensing rotation (skew angle) with a high sensitivity of 4.5 MHz/°. In another application, its dynamic part (groundless pattern) is used as the moving element, and hence, a displacement sensor is devised to detect the alignment. An extremely high sensitivity of 500 MHz/mm is recorded for a dynamic range of 1–5 mm. Moreover, the same stacked combination acts as a strain sensor, where the dynamic part is under stress. A range of 0–30 % stretch is found to deliver 12 MHz/% sensitivity on average. This unique design is shown to deliver all features of sensors suitable for tracking mechanical disturbances, and the measured results are congruent with the simulations.

## Figures and Tables

**Table 1:** Extracted parameters for the circuit of Figure 3**a.**

	*L* (nH)	*C* (pF)	*C_c_* (fF)	*L_s_* (nH)	*C_s_*(pF)
Single DSRR	2.94	0.3	21	2.72	0.3
Stacked DSRR	2.94	0.8	23	4.3	1.63

**Table 2:** Comparison of the performance of the sensors reported in literature and the proposed work.

.	Type	Freq. (GHz)	Q	Size ($\lambda_{g}\times \lambda_{g}$)	Disp.		Rotation (θ)		Stretch (s)	
					Range	$S_{disp.}$	Range	$S_{\theta }$	Range	$S_{s}$
[53]	Horn-Shaped	7	300^‡^	5.7 × 5.7	1–5	207	-	-	-	-
[54]	SRR	1.38	NA	1.75 × 1.08	-	-	0–8	1.87 dB/°	-	-
[45]	SRR	1.13	19^‡^	0.13 × 0.13	1.1	22.7	0	-	-	-
[44]	SRR	2.2–3.7	51^‡^	0.28 × 0.35^*^	0.3	65	0	-	_-_	-
[46]	SRR	1	129	0.08 × 0.08	-	-	0–90	0.25 dB/°	-	-
[47]	CSRR	2.03–3.63	4.5-4.8	2.5 × 3.5^*^	-	-	-	-	2.5–25	24
[55]	SRR	2.05–2.5	100^‡^	6.16 × 7.2^*^	0.5-3	108	-	-	-	-
**This Work**	DSRR	5.2	226	0.13 × 0.13	5	500	0–180	4.5 MH/°	0–30	12

‡: The stated values are approximated from figures. *: The principle of operation for these sensors contained more than one frequency and, for the sake of fair comparison, the size is evaluated based on their lowest resonance frequency.
